# A genomic resource derived from the integration of genome sequences, expressed transcripts and genetic markers in ramie

**DOI:** 10.1186/s12864-019-5878-8

**Published:** 2019-06-11

**Authors:** Yanzhou Wang, Zheng Zeng, Fu Li, Xiufeng Yang, Xinyue Gao, Yonghong Ma, Jing Rao, Hengyun Wang, Touming Liu

**Affiliations:** 1 Institute of Bast Fiber Crops and Center of Southern Economic Crops, Chinese Academy of Agricultural Sciences, Changsha, China; 2 Shanghai OE Biotech. Co., Ltd, Shanghai, China

**Keywords:** Ramie, Genome sequence, Transcriptome, Genetic map, Molecular marker

## Abstract

**Background:**

The redundancy of genomic resources, including transcript and molecular markers, and their uncertain position in the genome have dramatically hindered the study of traits in ramie, an important natural fiber crop.

**Results:**

We obtained a high-quality transcriptome consisting of 30,591 non-redundant transcripts using single-molecule long-read sequencing and proposed it as a universal ramie transcriptome. Additionally, 55,882 single nucleotide polymorphisms (SNPs) were identified and a high-density genetic map was developed. Based on this genetic map, 181.7 Mb ramie genome sequences were assembled into 14 chromosomes. For the convenient use of these resources, 29,286 (~ 95.7%) of the transcripts and all 55,882 SNPs, along with 1827 previously reported sequence repeat markers (SSRs), were mapped into the ramie genome, and 22,343 (~ 73.0%) transcripts, 50,154 (~ 89.7%) SNPs, and 1466 (~ 80.3%) SSRs were assigned to a specific location in the corresponding chromosome.

**Conclusion:**

This is the first study to characterize the ramie transcriptome by long-read sequencing, and the substantial number of transcripts of significant length obtained will accelerate our understanding of ramie growth and development. This integration of genome sequences, expressed transcripts, and genetic markers will provide an extremely useful resource for genetic, molecular, and breeding studies of ramie.

**Electronic supplementary material:**

The online version of this article (10.1186/s12864-019-5878-8) contains supplementary material, which is available to authorized users.

## Background

Ramie (*Boehmeria nivea* L. Gaud) is a diploid (2n = 28) species that has been cultivated for thousands of years as a fiber crop [1, 2]. Ramie fibers that are harvested from stem bark possess many excellent characteristics, such as smooth texture, long strands, and excellent tensile strength [3]. The length of these fibers can reach 55 cm, which is rare in the plant kingdom [4]. These excellent characteristics make ramie a widely cultivated crop in China, India, and other Southeast Asian and Pacific Rim countries. In China, ramie is the second most important fiber crop, and its total acreage and fiber production are second only to those of cotton [3].

Because of the significant economic importance of ramie, its agronomic traits have been widely targeted for genetic and molecular studies. To understand the expression profiling of ramie and identify important genes, the transcriptome of this crop has been de novo assembled in many studies [3, 5–15], resulting in the identification of millions of expressed transcripts. In addition, to characterize the genetic basis of traits, a total of 1827 and 2431 simple sequence repeat markers (SSRs) were developed in two previous studies [16, 17]. Based on these SSRs, 33 fiber yield-related quantitative trait loci (QTLs) and 29 flowering time-related QTLs were identified [18, 19]. In addition, 15 fiber yield-related QTLs detected from the population were genotyped by single nucleotide polymorphisms (SNPs) [20]. Recently, the ramie genome was sequenced and de novo assembled, providing an important basis for the genetic and molecular study of traits in this crop [21, 22].

Although plentiful genetic and genomic resources have been obtained from ramie, these resources are still inconvenient for researchers to use because there is considerable redundancy. Two sets of ramie SSRs were developed from the transcriptome by two independent studies [16, 17], causing potential redundancy of markers; undoubtedly, there were large numbers of redundant sequences among the transcripts identified from these transcriptomes [3, 5–15]. Furthermore, the absence of positional connections among the genome sequences, expressed transcripts, and molecular markers made them difficult to apply to other studies. For example, 33 and 15 fiber yield-related QTLs were mapped using SSRs and SNPs, respectively, in two previous studies [18, 20]. It is a challenge to identify the QTLs detected in these studies because the locations of the SSRs and SNPs in the chromosome were not determined. In order to integrate the information from genetic markers and the genome sequence, an integrated genetic and physical map was recently developed by assigning the genomic scaffold to chromosomes using a high-density genetic map; however, only 58.2 million bases (~ 17.3% of the ramie genome) were aligned to chromosomes [22].

To resolve this problem, we carried out single-molecule long-read sequencing to obtain a high-quality transcriptome and then identified the SNPs and developed a high-density genetic map using a segregated population. Thereafter, we integrated these new expressed transcripts and SNPs, along with the genomic sequences and SSRs reported previously [17, 21], into the chromosomes of ramie. These integrated resources will be an exceptionally valuable tool for many genetic, genomic, and breeding applications in this crop, such as QTL fine mapping and cloning and marker-assisted selection breeding.

## Results

### Single-molecule long-read survey of the ramie transcriptome

**Fig. 1:**
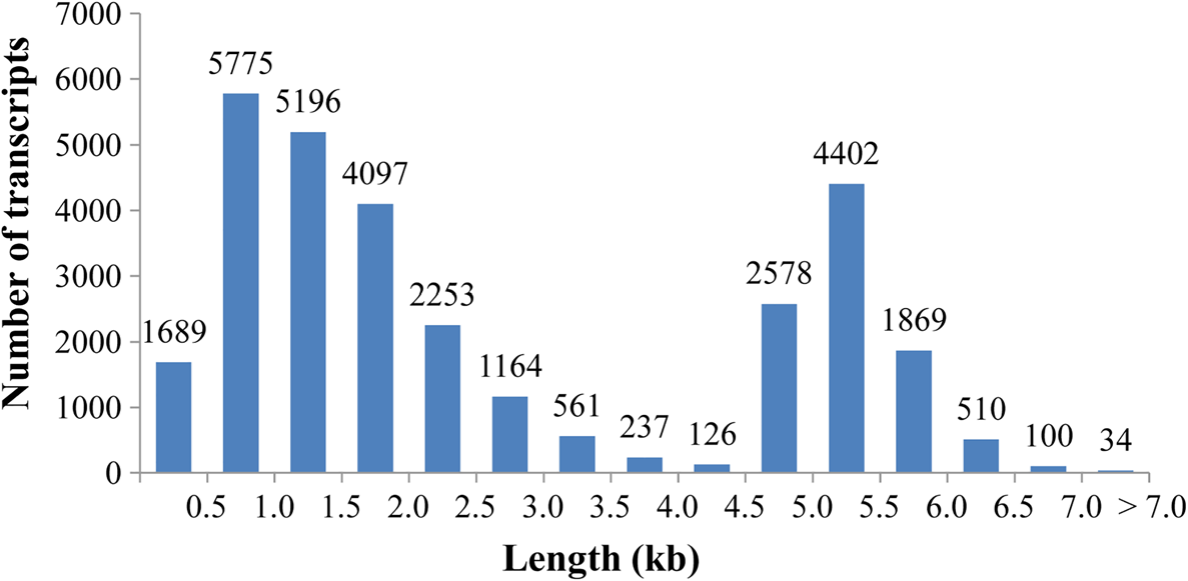
Length distribution of ramie transcripts generated from the single-molecule long-read sequencing

To obtain high-quality transcripts, the ramie transcriptome was sequenced using a single-molecule long-read sequencer from Pacific Biosciences. A total of 369,819 CCS reads representing 1.36 billion bases were generated, which resulted in a transcriptome consisting of 30,591 transcripts, accounting for 80.4 million bases in total (Table 1). Transcript length ranged from 299 bp to 11,307 bp, and the average length was 2629 bp (Table 1). These transcripts displayed a concentrated distribution in length in two ranges: 500–2500 bp (17,321; ~ 56.6%) and 4500–6000 bp (8849; ~ 28.9%); there were 13,834 (~ 45.2%) transcripts with a length of more than 2 kb (Fig. 1). Among these, 22,037 (~ 72.0%) and 27,816 (~ 91.0%) had sequences of 5′ UTR and 3′ UTR, respectively, indicating that most of these transcripts had a complete CDS sequence. In total 27,855 (~ 91.1%) transcripts achieved functional annotation (Table 1; Additional file 1: Table S1).

**Table 1:** Summary of ramie transcriptome determined by single-molecule sequencing

Number of circular consensus reads	369,819
Total bases of circular consensus reads (billion bases)	1.36
The size of transcriptome (Million bases)	80.4
Transcript number	30,591
Average length of transcript (bp)	2629
Transcript number annotated	27,855

### Assembly of genomic sequences into chromosomes

**Fig. 2:**
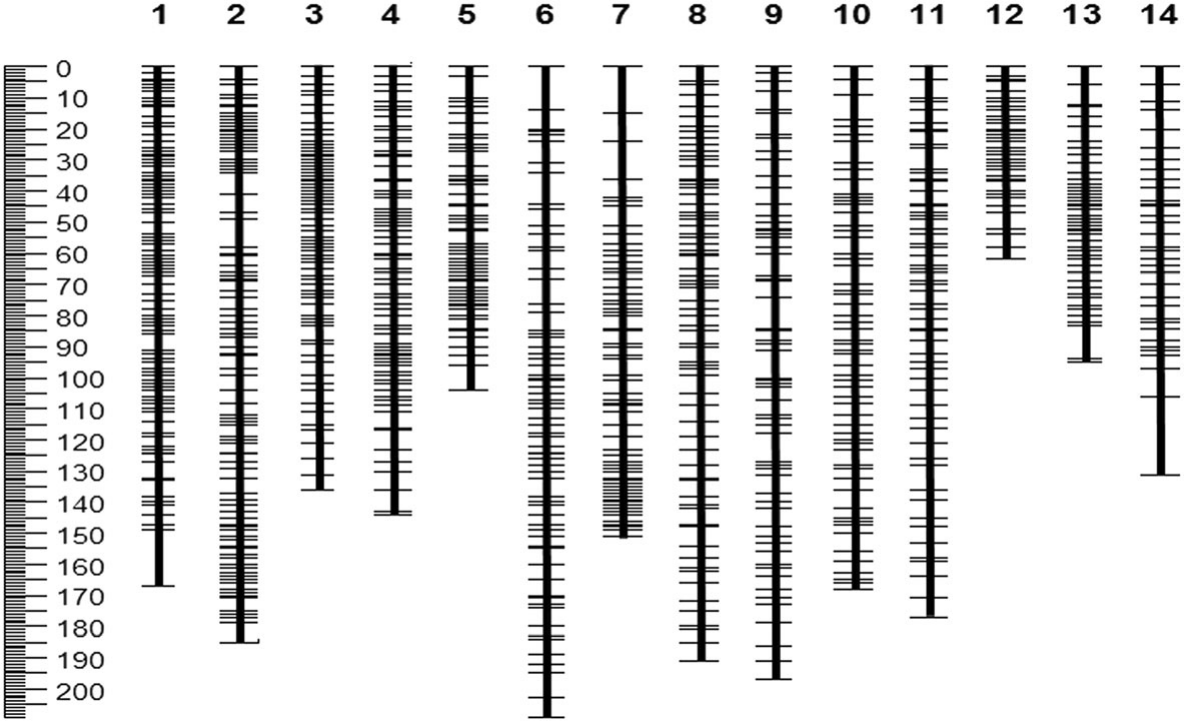
The high-density genetic map developed using the F_2_ segregated population derived from cultivated Zhongsizhu 1 and wild species *B. nivea* var. *tenacissima*

**Fig. 3:**
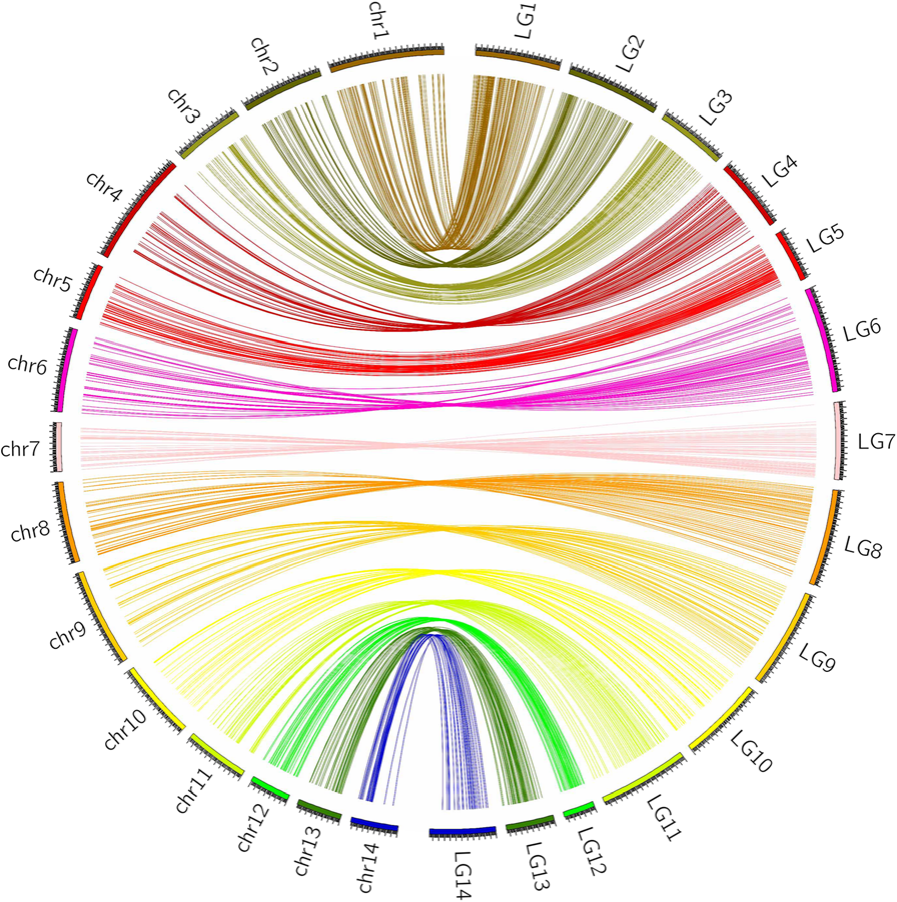
The chromosomes assembled by genomic sequences. A total of 170 scaffolds with a length of 181.7 Mb were assembled to the 14 chromosomes

The ramie draft genome, with a total length of 341.9 Mb, was de novo assembled by Luan et al. (2018). To anchor these sequences to the chromosome, we developed a high-density genetic map using a segregating population. In total, 55,882 high-quality SNPs were identified in this population (Additional file 2: Table S2; 10.6084/m9.figshare.8010446), of which 6194 showed polymorphisms with a segregation pattern of aa × bb between two parents. Thus, these 6194 SNPs were further used to develop the genetic map, which resulted in 14 linkage groups. The SNPs not assigned into linkage groups were filtered, and only one SNP was reserved as a bin marker when several SNPs were mapped into the same locus in the genetic map. Finally, a high-density genetic map consisting of 1085 SNPs was constructed, spanning a total length of 2118.8 cM (Fig. 2). The individual linkage groups contained binned markers ranging from 38 to 129 SNPs, with a length of 62.4–208.7 cM (Additional file 3: Table S3). Utilizing this genetic map, we assigned the genome sequences to the chromosome. The results showed that in total, 170 scaffolds with a length of 181.7 Mb (~ 52.7%; scaffold N50 = 2.98 Mb) were assembled into the 14 chromosomes (sequence file can be downloaded from 10.6084/m9.figshare.8010446), and the lengths assembled into individual chromosomes ranged from 7.1 Mb to 20.4 Mb (Fig. 3 and Table 2).

**Table 2:** Summary of genome sequences, expressed transcripts and genetic markers mapped into genome

	Genome sequence (Mb)	Transcripts	SNPs	SSRs
chr1	20.3	2214	5161	37
chr2	14.5	1917	3490	83
chr3	12.3	1492	3798	110
chr4	20.4	3325	6033	137
chr5	10.2	1242	2766	80
chr6	14.8	1880	3532	120
chr7	8.7	1079	2539	82
chr8	14.4	1584	3736	108
chr9	17.4	2008	4817	148
chr10	14.0	1824	3833	199
chr11	11.2	1316	3292	70
chr12	7.1	350	1176	63
chr13	8.0	957	2446	131
chr14	8.4	1155	3535	98
Total in chromosome	181.7	22,343	50,154	1466
Total in genome	–	29,286 (~ 95.7%)	55,882 (100%)	1827 (100%)

### Mapping transcripts, SNPs, and SSRs into the genome

**Fig. 4:**
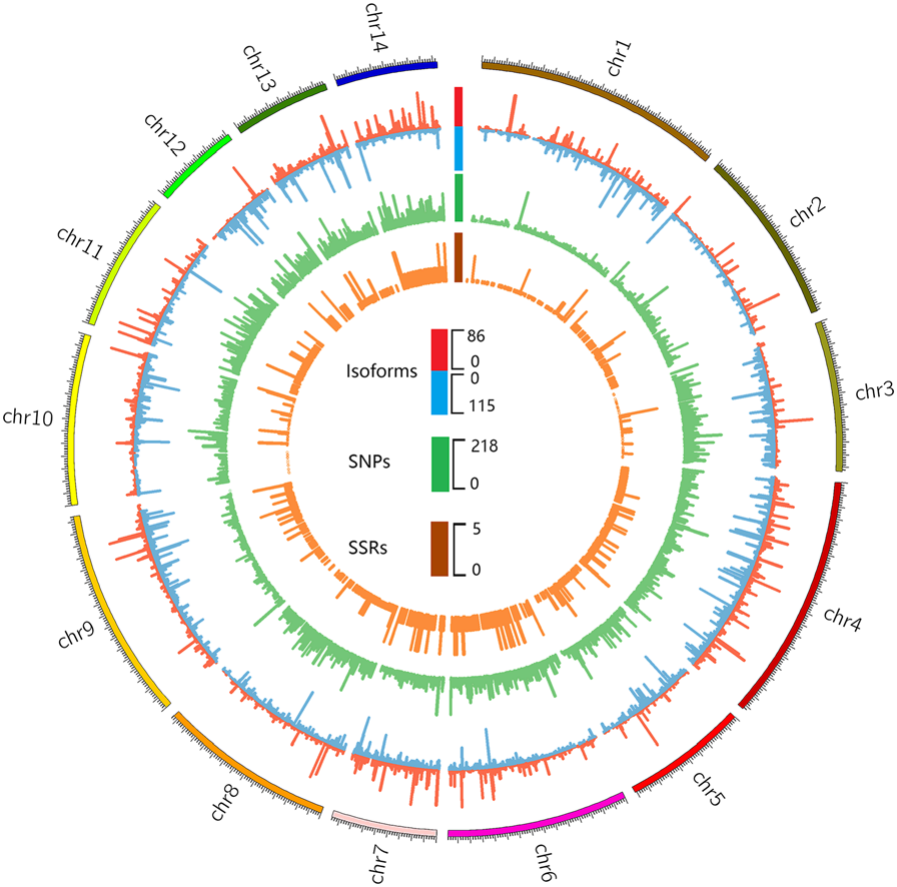
The distribution of expressed transcripts, SNPs and SSRs in 14 chromosomes. The outermost to innermost tracks indication the density of transcripts, SNPs and SSRs, respectively. The red and blue bars of transcripts’ track represent the densities of transcripts were transcribed from the positive and reversed DNA chain, respectively

To ascertain the location of 30,591 transcripts obtained from single-molecule sequencing, we mapped these transcripts into the genome. The results showed that in all 29,286 (~ 95.7%) were mapped into the genome, of which 14,489 and 14,797 were transcribed from the positive and reverse strand, respectively (Fig. 4; Additional file 1: Table S1). There were 22,343 transcripts (~ 73.0%) assigned to the corresponding chromosome. Interestingly, an extremely low density of transcripts was identified in chromosome 12 (only 49.3 per Mb), which was less than half the density identified in the other 13 chromosomes (Table 2).

Including the 1827 SSRs developed in our previous studies [17], a total of 55,882 high-quality SNPs were obtained in this study. We identified the location of these molecular markers in the genome and found that all SNPs and SSRs could be mapped into the genome. Of these, 50,154 (~ 89.7%) SNPs and 1466 (~ 80.3%) SSRs were assigned to specific location in the chromosome (Fig. 4; Additional file 2: Table S2; Additional file 4: Table S4). These markers were unevenly distributed in the ramie genome, with 37–199 SSRs and 1176–6033 SNPs per chromosome (Table 2).

## Discussion

### High-quality transcriptome obtained by single-molecule sequencing

The ramie transcriptome has been characterized in many previous studies, which have remarkably improved our knowledge about the growth and development of this crop [3, 5–15]. However, although a large number of transcripts were identified, in these studies, they were not always suitable for use in further research.. First, all the assembled transcriptomes were of low quality. There were more than 40,000 transcripts with an average length of ~ 800 bp, and only 49–77% of the transcripts could be used for functional annotation (Additional file 3: Table S5), which suggests that large numbers of these transcripts might be incomplete. In addition, ~ 411,000 transcripts were identified (Additional file 3: Table S5), whereas only 30,327 protein-coding genes were predicted from the ramie genome [21], indicating that many redundant transcripts were identified in the various transcriptomic studies, and that the same transcripts were likely assigned several different IDs across these studies. Therefore, it was essential to develop a high-quality transcriptome, with transcripts identified by a constant and uniform ID for use in future studies on ramie.

Unlike pair-end sequencing, which yields short reads, single-molecule PacBio sequencing can produce reads with an average length of over 10 kb and maximum read length of over 60 kb [23]. PacBio sequencing has been shown to be advantageous in characterizing the transcriptome owing to its ability to sequence full-length transcripts or fragments of significant length [23–25]. In this study, PacBio transcriptome sequencing resulted in the identification of 30,591 transcripts in ramie, with an average length of 2629 bp, and 91.1% of the transcripts were functionally annotated. Obviously, this was a significant improvement in both transcript length and the number of annotated transcripts as compared with previous studies [3, 5–15]. In addition, 72.0 and 91.0% of the transcripts had 5′ UTR and 3′ UTR sequences, respectively, which was far higher than in previous studies [7]. Therefore, compared to previous transcriptomes of ramie, the transcriptome we obtained from single-molecule PacBio sequencing was of superior quality, and we propose that it be used as a universal transcriptome for future studies on ramie. This is the first study to characterize the ramie transcriptome by long-read sequencing, and the substantial amount of transcripts of significant length obtained will accelerate our understanding on ramie growth and development.

### An integrated resource for ramie research

Genomic sequences, expressed tag sequences, and molecular markers are important tools for the genetic, molecular, and breeding study of crops. In model plants, these genetic and genomic resources are generally integrated into a platform, which makes them easier to use in basic and applied research [26, 27]. However, in ramie, although the complete genomic sequence has been obtained [21, 22] and large numbers of expressed sequences and SSRs have been identified from the transcriptome [3, 5–15], these resources are still difficult for researchers to use owing to the absence of positional connections among these resources. Taking a study on QTL cloning as an example, although a QTL was mapped into an interval flanked by two SSRs, the genomic sequences of this QTL region were unavailable because the positions of the SSRs were unknown in the genome. Therefore, for convenient application of these genetic and genomic resources, integration of these data is essential. In this study, the integration of genomic sequences, expressed transcripts, and molecular markers was carried out, resulting in ~ 95.7% of the transcripts and all investigated markers (including 55,882 SNPs and 1827 SSRs) being mapped into the ramie genome. In addition, ~ 52.7% of the genomic sequences were anchored to 14 chromosomes in order to construct a physical map. Although ~ 47% of genome sequences were not included in the chromosome, this physical map will still provide an important framework for further assembly of chromosome in the future. Additionally, ~ 73.0% of transcripts, ~ 89.7% of SNPs, and ~ 80.3% of SSRs were assigned to their corresponding chromosomes, indicating that most of the genes and genetic markers were included in this physical map.

## Conclusions

In conclusion, this study made a positional connection among genome sequences, expressed transcripts and genetic markers by integrating these resources, thus providing an extremely useful tool for future genetic, molecular, and breeding studies on ramie.

## Methods

### Single-molecule sequencing

Tissues of leaves, stems, roots, and flowers were collected from 30-day-old Zhongzhu 1 as a sample mixture and immediately frozen in liquid nitrogen. Total RNA of this sample was extracted using an E.Z.N.A. Plant RNA Kit (OMEGA Bio-tek, Norcross, GA, USA) according to the manufacturer’s protocol and used for single-molecule sequencing. In brief, approximately 1 μg of total RNA was used to construct an Iso-Seq SMRTBell library after a reverse-transcription reaction using a Clontech SMARTer cDNA Synthesis Kit (Clontech Laboratories, Mountain View, CA, USA) and oligo dT primer to generate full-length cDNA. Then, the library was subjected to single-molecule sequencing using a PacBio Sequel platform.

### Data analysis

Sequence data were processed using the SMRT analysis software (http://www.pacb.com/products-and-services/analytical-software/devnet/). Circular consensus sequence (CCS) reads were generated from the sub-read files using the default parameters. CCS reads were classified into four categories: full-length non-chimeric (FL), chimeric, non-full-length (non-FL), and short reads, according to whether the 5′ and 3′ primers and poly A were detected; if a read contained sequences of 5′ and 3′ primer and poly A, we deemed that the read was full-length. These full-length non-chimeric reads were clustered to obtain a consensus; only the clusters covered by at least 60 FL and non-FL reads were used to polish the consensus using the Quiver algorithm. Finally, redundant consensus isoforms were removed, and sequences that were non-redundant and unextended on either end were defined as transcripts.

### Transcriptome annotation

Transcripts were subjected to functional annotation by searching against six public databases, including the National Center for Biotechnology Information (NCBI) non-redundant protein sequences (NR), eukaryotic ortholog groups (KOG), Kyoto Encyclopedia of Genes and Genomes ortholog (KEGG), Swiss-Prot protein, Gene Ontology (GO), and Interpro databases [28]. The E-value cutoff for the annotated search was set to 10^− 5^. The coding sequence (CDS) of each transcript was predicted by BLAST search against the NR database (https://blast.ncbi.nlm.nih.gov/Blast.cgi) and the SwissProt protein database (http://www.uniprot.org/uniprot/), as well as by using the ESTscan program [29]. The 5′ UTR and 3′ UTR sequence for each transcript was identified by aligning the CDS into the corresponding transcript.

### Construction of a genetic map

A population consisting of 111 F_2_ progenies derived from cultivated Zhongsizhu 1 and wild species *B. nivea* var. *tenacissima* was used for construction of the genetic map. To detect the SNPs, the clean reads sequenced from the transcriptome of 111 F_2_ families and two parents by Illumina sequencing (SRA accession no. SRP182925) were aligned into the ramie genome (accession ID: PHNS00000000) [21] using Burrows-Wheeler Aligner (BWA) software (version: 0.7.17; settings: mem –t − 4 –k 32 –MR) [30], and then the alignment files were converted to bam files using SAMtools (settings: –bS –t) [31]. If there were multiple read pairs mapped to the same position or with external coordinates, only the pair with the highest mapping quality was retained. Thereafter, SNP discovery was performed for the two parents and 111 families using SAMtools [31]. Because the two parents were heterozygous, the polymorphic markers between them were classified into eight segregation patterns: ef × eg, nn × np, ab × cc, aa × bb, ab × cd, lm × ll, hk × hk, and cc × ab [20, 32, 33], according to the CP model in JoinMap 4.0 [34]. The numbers of SNPs, transitions, and transversions were also counted, and a Perl script (uploading in 10.6084/m9.figshare.8010446) was used to filter out the SNPs with more than two genotypes, retaining only polymorphic markers with the ‘aa × bb’ segregation pattern. Finally, retained markers that contained abnormal bases or exhibited significantly distorted segregation (*P* < 0.001) or non-integrity (missing data in > 30% progenies) were filtered out using JoinMap 4.0 [35]. The regression algorithm, three circulation sequences, and Kosambi mapping function were used for marker distance calculation [35], and the linkage map was drawn using mapchart 2.32 [36], with the default parameters.

### Anchoring genomic scaffolds to the genetic map

Sequences of ramie genome assembled by Luan et al. [21] were anchored into the chromosome using SNP markers localized on the genetic map by the following method. First, all of the markers were aligned against the genomic scaffolds using BLASTN with an E-value threshold of 10^− 15^. Markers that were mapped to more than one scaffold were discarded. Then, the scaffold order on the chromosome was sorted by the mean value of markers on the same scaffold. The physical order of the markers and their linkage map order were compared to determine the orientation of the scaffold on the chromosome. If the number of markers in a scaffold that had the same order in both the physical and linkage map order was greater, the scaffold was considered to have a positive orientation; otherwise, the orientation of the scaffold was negative and the sequence was reverse-complemented. Scaffolds in which the orientation was unknown remained in a positive orientation by default. Finally, a visualized map of linkage groups that correlated with anchored scaffolds was constructed and exported to create a physical map.

### Mapping the transcripts, SNPs, and SSRs into the genome

To ascertain the position of transcripts obtained by single-molecule sequencing in this study, all transcripts were aligned into the genome by GMAP software [37], using default parameters. Thereafter, the SAMtools [31] and BEDtools [38] were used to identify the positional information, including the scaffold ID and the position in the scaffold. Finally, the position of the transcript in the chromosome was ascertained based on the anchoring of the scaffold into the chromosome. In addition, the chromosomal locations of the SNPs discovered in this study were identified by aligning these SNPs into the genomic scaffolds anchored to the chromosome using GMAP software [37] with default parameters. We developed 1827 SSRs in our previous study, and their genomic locations were determined using the following method. First, the forward and reverse primers of SSR were used to perform BLASTN analysis, and if both primers showed a complete match to an adjacent region in a scaffold (< 400 bp), this region was considered a SSR loci, and the SSR locations in the genome were determined based on the corresponding scaffold anchored into the chromosome. The distribution of transcripts, SNPs, and SSRs was visualized by a circos program [39].

## Additional files

Additional file 1: **Table S1.** Annotation and position information of 30,591 transcripts obtained from single-molecule sequencing. (XLSX 5634 kb)

Additional file 2: **Table S2.** Information of 55,882 SNPs. (XLSX 3106 kb)

Additional file 3: **Table S3.** Summary of the high-density genetic map. **Table S5**. Basic information of transcriptomes de novo assembled by previous studies. (DOC 46 kb)

Additional file 4: **Table S4.** Information of 1827 SSRs. (XLS 530 kb)

## Data Availability

The CCS reads from the single-molecule sequencing effort are available in the NCBI SRA database under the accession number SRR8490047. The transcriptome sequence can be downloaded from the NCBI GenBank database under the accession number GHEV00000000.

## Abbreviations

CCS: Circular consensus sequence
CDS: Coding sequence
QTL: Quantitative trait locus
SNP: Single nucleotide polymorphism
SSR: Sequence repeat marker
