# Supplementary material for: A genomic resource derived from the integration of genome sequences, expressed transcripts and genetic markers in ramie
Source: BMC Genomics. 2019 Jun 11;20:476. doi: 10.1186/s12864-019-5878-8 (PMC6558782; doi:10.1186/s12864-019-5878-8)
Supplement: Supplementary file 3 — Table S3. Summary of the high-density genetic map. Table S5. Basic information of transcriptomes de novo assembled by previous studies. (DOC 46 kb) [file 12864_2019_5878_MOESM3_ESM.doc]

Table S3 Summary of the high-density genetic map

| Linkage group | Number of markers | | Length (cM) | | Average marker interval (cM) | | Maximum interval (cM) | | Number of interval with > 10 cM | |
| --- | --- | --- | --- | --- | --- | --- | --- | --- | --- | --- |
| LG 1 | 129 | 167.1 | | 1.31 | | 17.81 | | 1 | |  |
| LG 2 | 113 | 184.8 | | 1.65 | | 8.45 | | 0 | |  |
| LG 3 | 103 | 136.0 | | 1.33 | | 5.41 | | 0 | |  |
| LG 4 | 84 | 144.4 | | 1.74 | | 7.48 | | 0 | |  |
| LG 5 | 81 | 104.4 | | 1.31 | | 7.93 | | 0 | |  |
| LG 6 | 77 | 208.7 | | 2.75 | | 14.42 | | 2 | |  |
| LG 7 | 71 | 152.2 | | 2.17 | | 15.50 | | 2 | |  |
| LG 8 | 71 | 190.9 | | 2.73 | | 6.16 | | 0 | |  |
| LG 9 | 66 | 196.7 | | 3.03 | | 9.74 | | 0 | |  |
| LG 10 | 66 | 167.8 | | 2.58 | | 8.38 | | 0 | |  |
| LG 11 | 65 | 177.4 | | 2.77 | | 7.81 | | 0 | |  |
| LG 12 | 63 | 62.4 | | 1.01 | | 4.63 | | 0 | |  |
| LG 13 | 58 | 95.4 | | 1.67 | | 10.07 | | 1 | |  |
| LG 14 | 38 | 130.6 | | 3.53 | | 24.81 | | 1 | |  |
| All | 1085 | 2118.8 | | 1.98 | | 24.81 | | 7 | |  |

Table S5 Basic information of transcriptomes *de novo* assembled by previous studies

| Varieties | Transcript number | Average length (bp) | Transcripts annotated | Reference |
| --- | --- | --- | --- | --- |
| Zhongzhu 1 | 43,990 | 824 | 34192 (77.7%) | Liu et al. 2013 |
| Zhongzhu 1 | 50,486 | 853.3 | 24820 (49.2%) | Zhu et al. 2014 |
| Qingyezhuma | 56,932 | 817.4 | 33265 (58.4%) | Liu et al. 2015 |
| Huazhu No. 5 | 43,222 | - | - | Huang et al. 2014 |
| Zhongzhu 1 | 70,721 | - | 36,535 (51.7%) | Al-Ani and Deyholos 2018 |
| 1504 | 58,369 | - | - | Chen et al. 2014 |
| Chuanzhu 8 | 46,533 | 845 | 24,327 (52.3%) | Zeng et al. 2016 |
| Qingdaye | 40,826 | 830 | 26,851 (65.8%) | Yu et al. 2015 |
